# A spatial reconnaissance survey for gold exploration in a schist belt

**DOI:** 10.1016/j.heliyon.2021.e08406

**Published:** 2021-11-15

**Authors:** Andongma W. Tende, Mohammed D. Aminu, Abdulgafar K. Amuda, Jiriko N. Gajere, Hadiza Usman, Fatima Shinkafi

**Affiliations:** a Department of Geology, Kano University of Science and Technology Wudil, Kano, Nigeria; b Department of Petroleum Chemistry, American University of Nigeria, Yola, Nigeria; c Department of Geology, Bayero University Kano, Kano Nigeria; d Department of Geology, Nasarawa State Polytechnic, Lafia, Nigeria; e Atiku Institute for Development, Yola, Nigeria; f Solid Minerals Development Fund, Abuja, Nigeria

**Keywords:** Gold, Mineral deposit, Schist belt, Malumfashi, Predictive modelling

## Abstract

Geological data integration and spatial analysis for structural elucidation are more assertive approaches for reconnaissance scale mineral exploration. In this study, several methods involving Fry analysis, distance correlation analysis, prediction area plots as well as knowledge driven predictive models including TOPSIS, ARAS and MOORA were systematically employed for unravelling the spatial geological attributes related to gold mineralisation. Additionally, statistical validation of knowledge driven predictive models were implemented using the Receiver Operating Characteristic/Area Under Curve analysis (ROC/AUC). The evidence from Fry and distance correlation analysis suggests that gold occurrence within parts of the Malumfashi schist belt of Nigeria is defined by a strong spatial association with the ENE-WSW as well as the NNE-SSW trending structures. The prediction area plot also revealed a robust spatial correlation between mineral occurrence and spatial data related to geological structures. The application of knowledge driven predictive models suggest a high favourability for gold occurrence within the southern, central, and north-eastern parts of the study location, while statistical validation using the ROC/AUC curves suggest a high prediction accuracy greater than 70% for all models. The geospatial analysis for mineral exploration within the Malumfashi area has unveiled an invaluable geological criterion for gold targeting with a considerable level of certainty.

## 1 Introduction

Mineral exploration remains an integral part of geological investigations as it attempts to unravel the most favourable zones for possible occurrence of economic deposits. In many cases, several methods consisting of geochemistry, mineralogy, petrology, geophysics, and remote sensing/Geographic Information System (GIS) are employed for feasibility assessment and target delineation of mineral resources across a wide range of geological environments [1, 2, 3, 4, 5, 6, 7, 8].

The choice of exploration method employed is usually dependent on the ore deposit character, its geological attributes and more specifically, the scale of geological prospecting. Although geochemistry and geophysical methods have been widely used for mineral exploration, recent developments from remote sensing/GIS approaches have been invaluable for exploring a wide range of mineralisation across numerous mineral belts around the world [9, 10, 11].

On a regional scale, the use of remote sensing and GIS remains the best alternative as it has been credited with enormous success with respect to time efficiency, prediction accuracy and ability to explore inaccessible areas. The practical applications of remote sensing/GIS methods to mineral exploration are often centred on three main aspects consisting of satellite mapping of hydrothermal alteration, structural investigation of ore geometry and spatial data integration for prospectivity map development [12, 13, 14, 15]. Amongst these methods, the spatial data integration of geological dataset remains the most incorporative and highly comprehensive as it attempts to capture geological information from diverse sources.

The implementation of data integration techniques is generally based on sufficient knowledge of mineral deposit evidence. According to Carranza [16], the application of knowledge driven predictive models are usually more suitable for less explored zones associated with insufficient information pertaining to mineral occurrence. On the other hand, spatial exploration of terrains with significant evidence for mineral occurrence are more suitable for data driven integration methods, which is often aimed at identifying spatial patterns identical to zones of mineral occurrence. In Nigeria, the practical use of remote sensing and GIS technology for exploration purposes are limited. This could be attributed to limitations on technological advancement and absence of reliable data or software. Most researchers within this scope often try to elucidate the enormous potential of remote sensing by investigating a wide range of mineral deposits using different remote sensing/GIS methods [17, 18, 19].

In exploring for gold within the Nigerian basement complex, remote sensing/GIS dataset remains the most suitable technique owing to its ability to map and delineate hydrothermal alterations as well as geological structures associated with gold occurrence [20, 21, 22]. Also, gold can be preferentially mapped using geophysical dataset such as aeromagnetic or radiometric data [23, 24]. In this study, we attempt to predict favourable zones for gold mineralisation within parts of the Malumfashi schist belt by applying optimised knowledge driven methods such as TOPSIS, ARAS and MOORA. Although TOPSIS has previously been applied to mineral exploration surveys [25, 26, 27], the application of ARAS and MOORA methods are relatively new.

## 2 Geological setting

**Figure 1:**
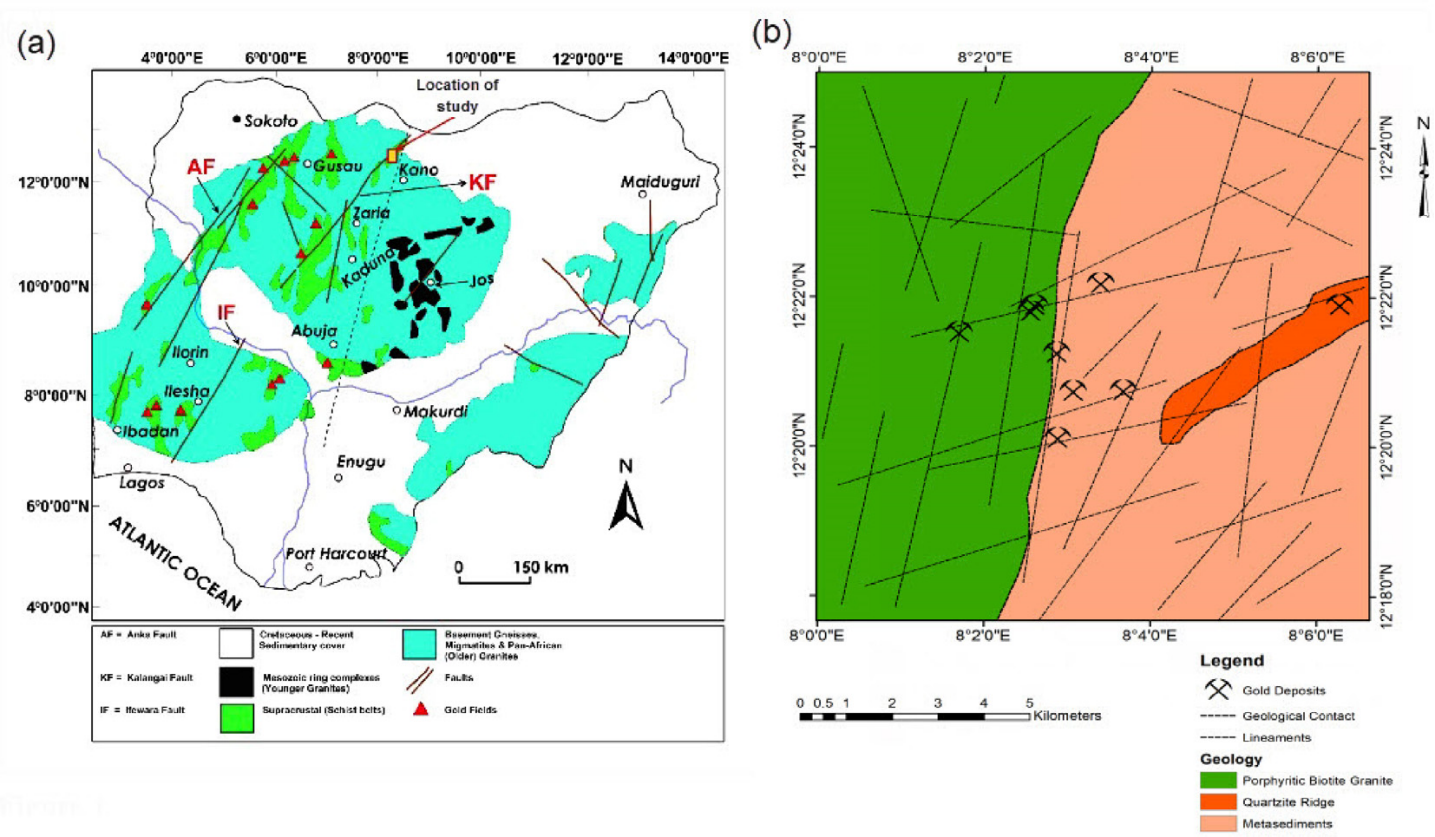
(a) Regional geology of the Nigerian Pan-African basement, showing major faults, Younger Granites and location of study area, modified after Woakes et al. [32]; (b) Simplified geological map of the north-eastern edge of Malumfashi schist belt adopted from Andongma et al. [33].

The Nigerian basement complex forms a part of the Pan-African mobile belt and lies between the West African and Congo Cratons, and south of the Tuareg Shield (Figure 1a) [28]. The collision at the plate margin is believed to have led to the reactivation of the internal region of the belt [29]. Ferré and Caby [30] noted that the rocks formed from at least four major orogenic cycles corresponding to Liberian (2,700Ma), Eburnean (2,000 Ma), Kibaran (1,100 Ma), and Pan-African cycles (600 Ma). The cycles were characterized by intense deformation and isoclinal folding accompanied by regional metamorphism, which was further followed by extensive migmatisation. The Late stages of Pan-African orogeny are characterized by emplacement of granites and granodiorites and associated contact metamorphism [31].

The basement complex consist of three main lithological units including the migmatite gneiss complex, low grade metasedimentary cover and syn-tectonic to late tectonic granitic intrusions (Figure 1b) [29]. According to Rahaman [34], the migmatite gneiss complex is generally considered as the basement *sensu stricto* accounting for about 30% of the total surface area of Nigeria. It constitutes heterogeneous assemblages of rock suits including the migmatites, gneisses of various origin and basic and ultra-basic rocks such as amphibolite and talc schists [35]. The occurrence of metasedimentary relics within the migmatite gneiss complex have been classified by Oyawoye [36] as ancient metasedimentary formations, however, low grade metasedimentary units are commonly observed within the western half of the basement complex characterized by extensive N–S trending schist belts [37]. The lithological attributes of younger metasediments are essentially psammitic to pelitic sediments of low grade and occasionally conglomeritic facies interbedded into lavas. The older granites or Pan-African granitic rocks are known to have intruded both the schist and migmatite gneiss complex [35], consisting of rock units which include granite, tonalite and charnockite of varying composition [38]. Radiometric dating of the older granites have yielded ages corresponding to 750-500 Ma [39].

The present study area lies within the Malumfashi schist belt (Figure 1). It covers an area of about 165 km^2^ between latitudes 12⁰ 18ʹ and 12⁰ 24ʹ 30ʹʹ N and longitudes 8⁰00ʹ and 8⁰ 6ʹ 30ʹʹ E (Figure 1b), and has an average elevation of about 580 m above sea level. McCurry [40] and Andongma *et al.* [33] provided a detailed description of the petrology of the Malumfashi schist belt (Figure 1b). It mainly comprises of gneisses, metasediments that were intruded by Pan-African granitoids. Structurally, the axial planes of schists trends NNE-SSW with tight to isoclinals D_1_ fold. Also, early foliations related to D_2_ folds and the traces of older deformation episodes which trends ENE-WSW are largely obliterated [40].

**Figure 2:**
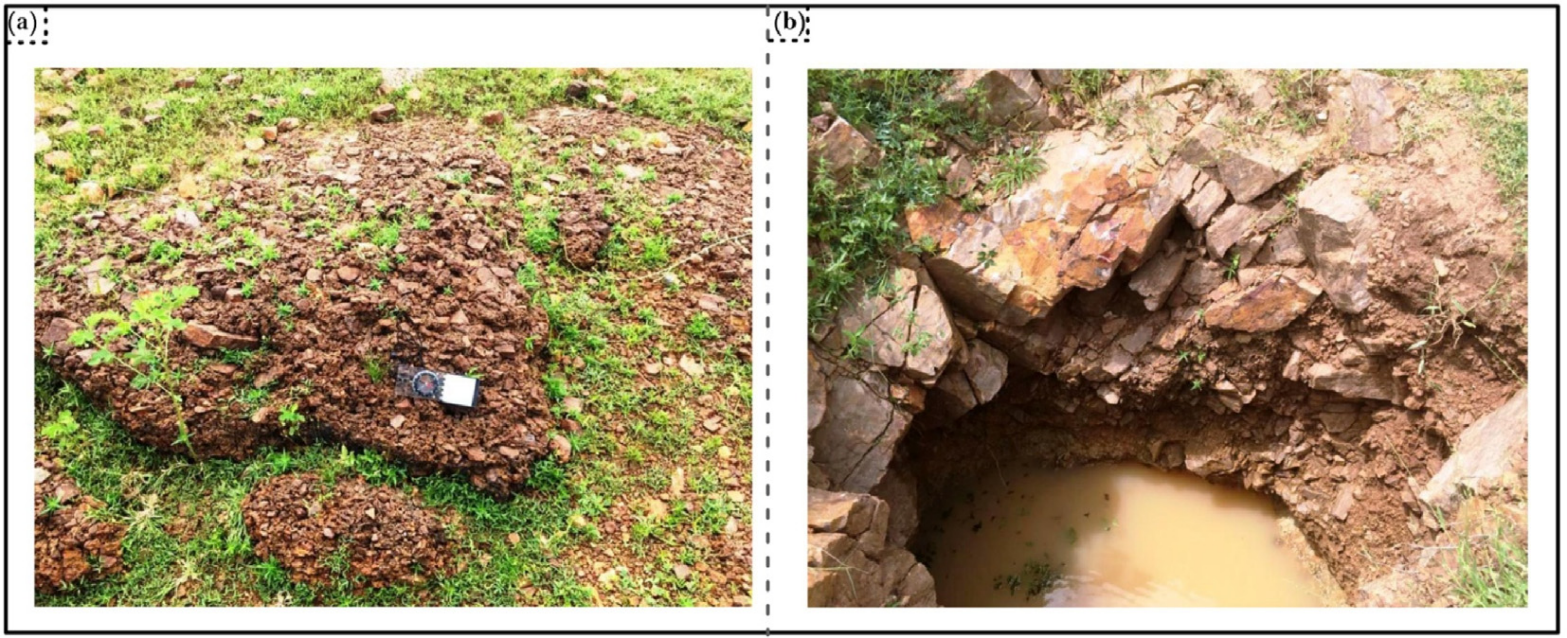
Field photograph of low-lying (a) Lateritic cover on schist; (b) Quartz-schist (metasediment) whose marginal contact is dug for alluvial gold exploration.

The schists are covered by thick laterites, like gossans (Figure 2a). The panning of sediments around the laterites and decomposed apical parts of host rocks yield gold grains with morphology akin to grains that are close to its primary origin. Artisans also dig pits around quartz and schist margins to explore alluvial gold (Figure 2b).

## 3 Materials

### 3.1 Target data

Information on gold occurrence within the study location were collected by a concise field survey. GPS co-ordinate locations were obtained using a Garmin eTrex® 10 GPS receiver and then transferred onto Microsoft Excel spreadsheet program where they were converted into point shape using ArcGIS 10.3 software.

### 3.2 Exploration data

In this study, spatial data used for gold prospectivity analysis include:

• Ferric iron alteration intensity
• Ferrous iron alteration intensity
• Proximal distances to host rock
• Lineament density
• Proximal distances to ENE-WSW lineaments
• Proximal distances to NNE-SSW lineaments
• First vertical derivatives (FVD)

A basic summary of the spatial data used in gold predictive analysis is illustrated in Table 1. Spatial data on ferric and ferrous iron distribution across the study location were obtained from a digital processed Landsat Enhanced Thematic Mapper (ETM) imagery downloaded from USGS [41]. Basically, the Landsat ETM data consist of 8 bands, including two visible, two very near infrared, two shortwave infrared, one thermal infrared and one Panchromatic band. A well-prepared band ratio 5/4 and 3/1 were invaluable in evaluating the spatial distribution of ferric and ferrous iron alterations within the study location. Information on lithological units for the study location was subset from a regional geological map of Nigeria published by the Nigerian Geological Survey Agency (NGSA [42]).

**Table 1:** Summary of spatial data used in mineral predictive analysis.

	Primary Data	Source	Spatial Data	Recognition criteria
1	Landsat ETM	USGS [41]	Band ratio 5/4	Enhanced zones of ferric iron alteration
2	Landsat ETM	USGS [41]	Band ratio 3/1	Enhanced zones of ferrous iron alteration
3	Regional geology of Nigeria	NGSA [43]	Euclidean distance to host rock	Proximal distances to host rock
4	Digital elevation Model	USGS [41]	Lineament density	Zones of high lineament density
5	Digital elevation Model	USGS [41]	Euclidean distances to NNE-SSW lineaments	Proximal distances to NNE-SSW lineaments
6	Digital elevation Model	USGS [41]	Euclidean distances to ENE-WSW lineaments	Proximal distances to ENE-WSW lineaments
7	Total magnetic Intensity	NGSA [43]	First vertical derivative analysis	High magnetic anomalies

The study area is underlain by lithologies of Pan-African granites and metasedimentary rocks as shown in Figure 1. The gold occurrence within the study area is hosted within rocks of metasedimentary units. A well processed spatial data on distances to host rock (distances to metasedimentary rock units) was prepared by the application of Euclidean distance analysis. Information on geological structures were obtained by manual digitisation of linear features observed on digital elevation model [44, 45] which was downloaded from USGS [41].

**Figure 3:**
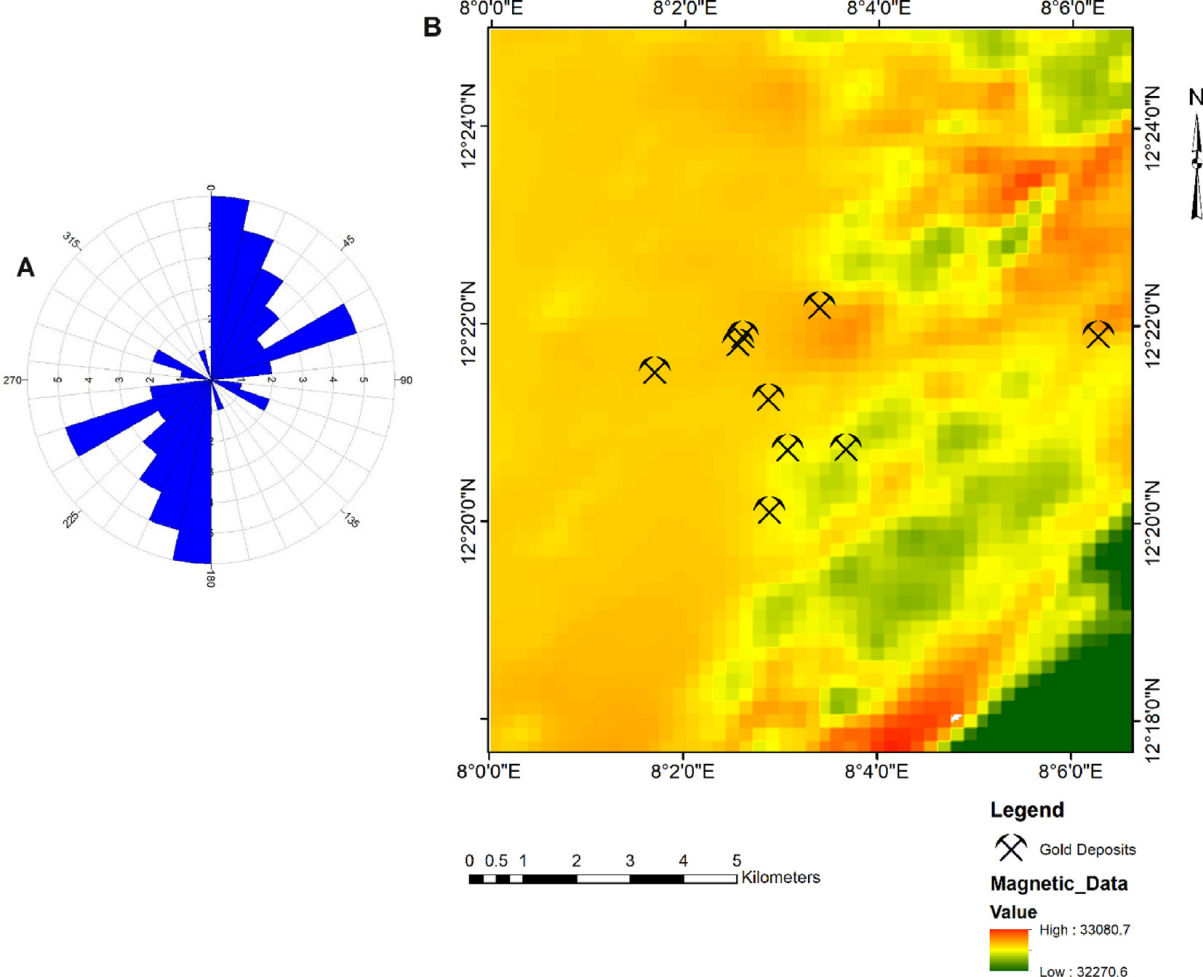
(a) Rose plot for digitally extracted lineaments; (b) Total Magnetic Intensity data for the study location.

The digitized lineaments shown in Figure 1, is characterised by a spatial distribution of the NNE-SSW and ENE-WSW lineament patterns. A rose plot for digitised lineaments suggests the prevalence of N–S trending lineaments accompanied by a subtle ENE-WSW lineament trend (Figure 3a). The application of kernel density algorithm facilitated the generation of lineament density data for the study area. Information on proximal distances to favourable geological structures were prepared by the application of Euclidean distance analysis to the NNE-SSW and ENE-WSW trending lineaments. Geophysical data consisting of Total Magnetic Intensity (TMI) imagery (Figure 3b) was digitally filtered by application of first vertical derivative algorithms. The TMI data obtained from the NGSA [42] forms part of the regional aeromagnetic data acquired for NGSA between 2004 and 2009 by Fugro Airborne Surveys Limited. Magnetic data was acquired at a flight height of 80 m on a series of NW-SE trend perpendicular to the regional trend with flight lines spaced and tied at 500 m and 200 m respectively. Data was effectively corrected for diurnal variations and the main component of the geomagnetic field removed.

### 3.3 Conceptual model and exploration criteria

**Figure 4:**
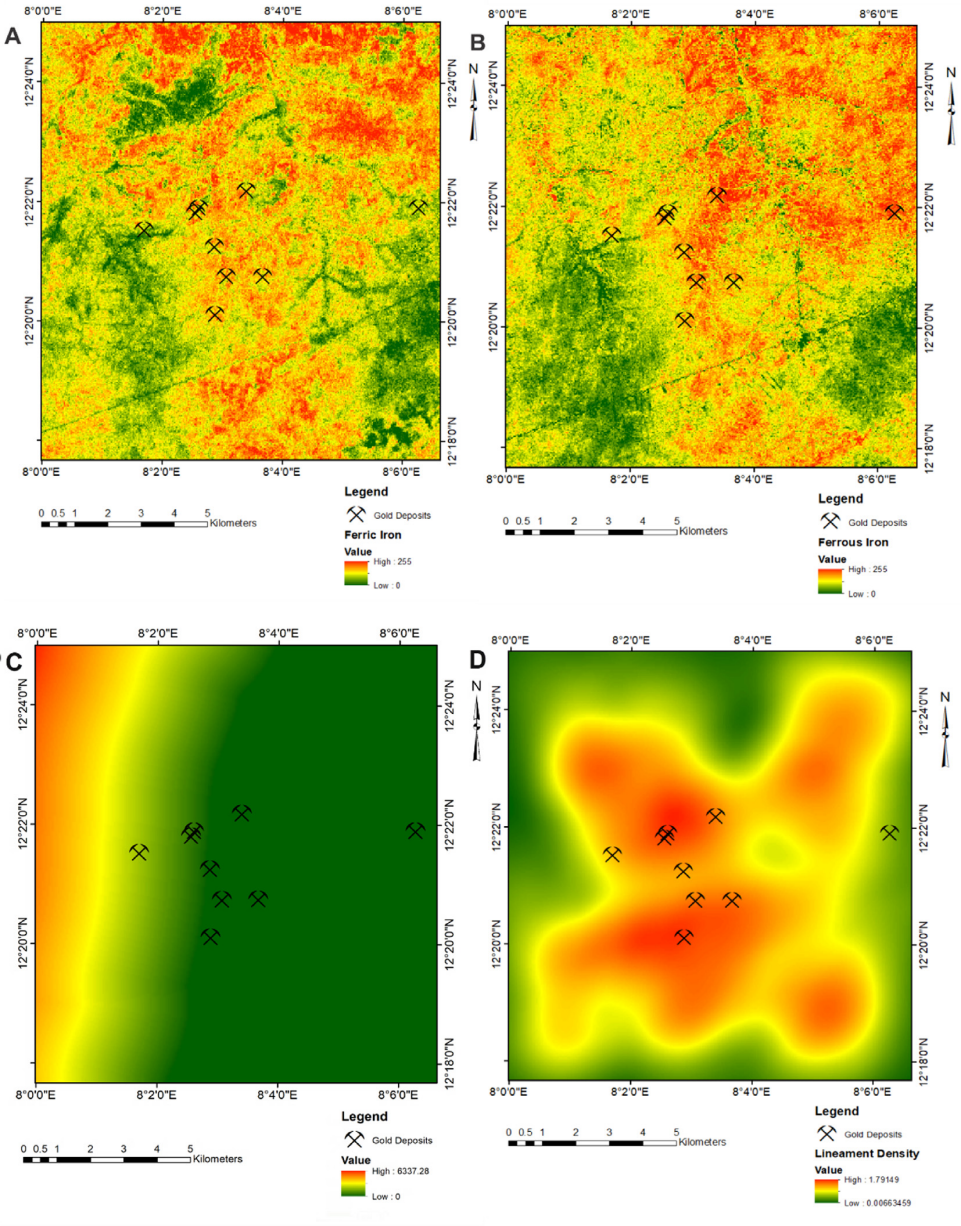
Spatial exploration datasets: (a) Ferric iron; (b) Ferrous Iron; (c) Lithological Data; (d) Lineament Density.

Gold occurrence within the Nigerian schist belts are mesothermal, with origin linked to metamorphic processes of devolatilization and dehydration of subducted crustal materials along a collisional sutured zone [46]. The hydrothermal systems associated with these deposits are generally widespread and represents a regional fluid typical of inherent tectonism along convergent margins [47]. According to Garba [48], the gold mineralisation in Nigeria commonly occur with pyrite, chalcopyrite, galena and minor amounts of sphalerite, magnetite and intergrowth of quartz and quartz carbonates. The chemical significance of Fe in gold mineralization arises from its ability to react with sulphur in solution to deposit gold. Thus, a well-prepared satellite derived alteration map of iron alteration (Figure 4a and b) is highly relevant for identifying potential sites for gold deposits. On these images, zones of high iron alterations represented by red pixels were considered more favourable for identifying gold related mineralization. The geological influence of host rock on gold mineralisation can be explain in terms of its mechanical and chemical attributes [49, 50]. Generally, the presence of a potential host rock in combination with low tensile strength and either elevated Fe or C are favourable attributes for precipitation of gold mineralisation [51].

**Figure 5:**
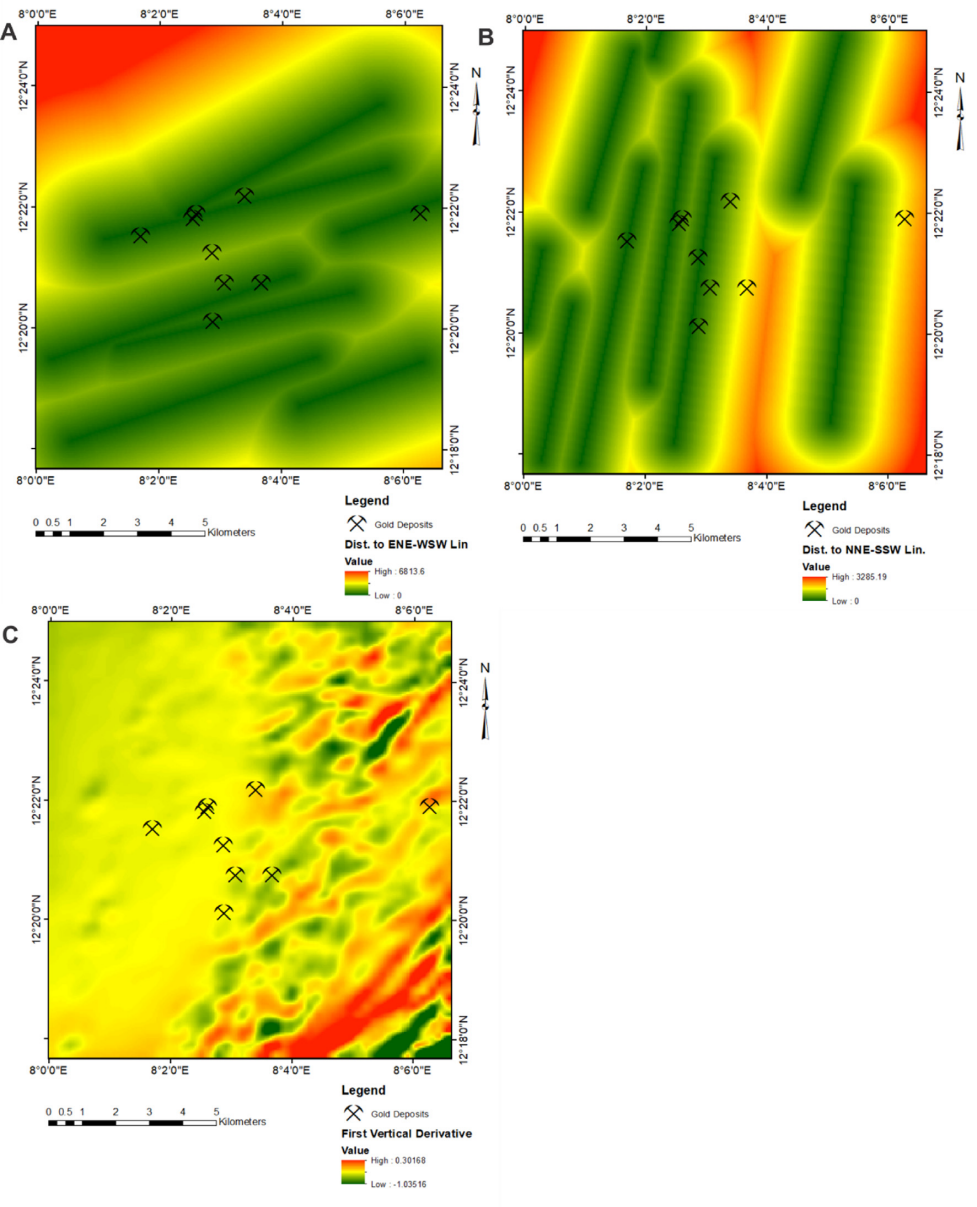
Spatial exploration datasets: (a) Distances to ENE-WSW lineaments; (b) Distances to NNE-SSE Lineaments; (c) First vertical derivative.

In Nigeria, metasedimentary rocks are generally considered the most viable lithological units for gold mineralisation [20, 52]. By implication, zones that are proximal to the existing host rock represented by green pixels are considered more suitable exploration targets for gold mineralisation. A well-prepared spatial data on proximal distances to host rock (Figure 4c) may serve as a valid spatial data for gold exploration. Geological structure is arguably an influential parameter necessary for formation of gold mineralisation, and a high fracture density within fault zones may provide suitable pathways of enhanced permeability and fluid flow during metamorphism [53]. A well-prepared exploration data on lineament density (Figure 4d) is highly relevant for identifying zones of enhanced permeability. Thus, zones of high lineament density represented by red pixels were considered as favourable target for gold exploration. Gold mineralisation within Nigeria is confined to several regional faults system. Field studies and evidence within the Malumfashi area suggest that gold occurrence are more affiliated to the NNE-SSW and ENE-WSW trending directions. A distance to favourable geological structures (Figure 5a and b) are valid exploration data for gold prospecting. On these images, proximal distances to favourable geological structures (represented by green pixels) were more intrinsic for exploring gold mineralisation.

The environmental dynamics associated with rock magnetism validates the use of aeromagnetic data for gold prospecting [54]. Usually, high anomalies on magnetic data are attributed to the presence of magnetite and in most mineral systems, hydrothermal fluids are known to degrade magnetite concentrations, resulting to demagnetisation of anomalies [55]. High magnetic anomalies in metasedimentary rocks may be attributed to several factors among which are the presence of residual magnetite within lateritic materials [56]. In the study area, gold occurrence are closely associated with weathered lateritic bodies yielding a positive anomaly on aeromagnetic data. On the first vertical derivative magnetic data (Figure 5c), zones of high magnetic intensities represented by red pixels were considered more favourable for gold prospecting.

## 4 Methods

**Figure 6:**
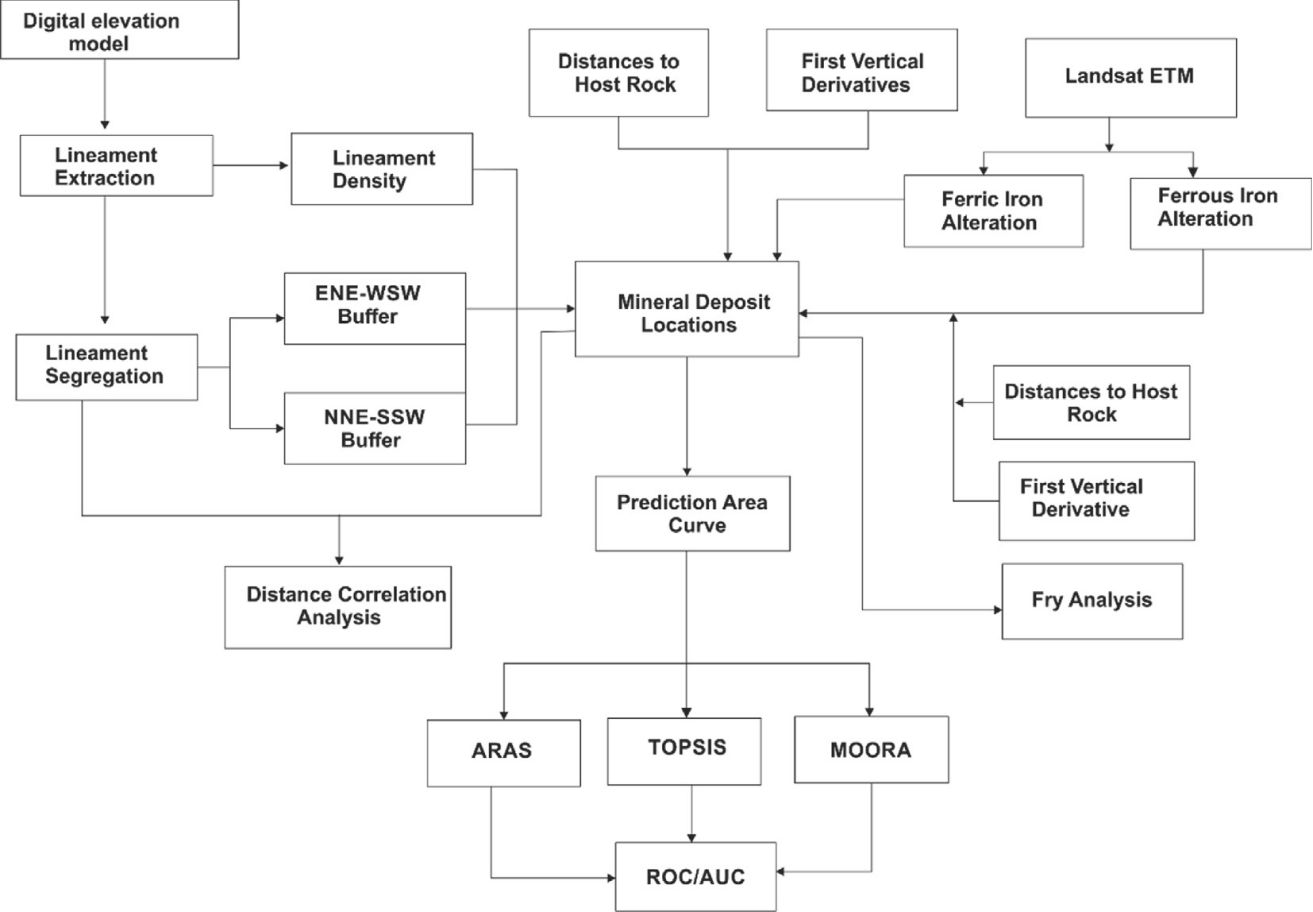
A summarised flow chart for the study.

A spatial illustration of methods used in the study is shown in Figure 6. The description of every parameter is explained accordingly.

### 4.1 Fry analysis

The detailed information on directional anisotropy of spatial distribution of mineral deposits at different scales can be obtained using Fry analysis [57]. The Fry analytical method can be described as a geometrical technique of spatial auto correlation analysis of point objects [58, 59]. It is best for determining direction(s) of maximum continuity and can be applied in the study of different mineral deposits at scales [60]. Development of Fry plots was carried out using a proposed manual method [61]. In order to develop Fry plots, mineral deposit locations were carefully plotted on a map and a series of parallel reference lines drawn. A tracing paper with origin (central point) and set of parallel lines were then superimposed on the first such that the origin coincides with one of the mineral deposit locations. On the tracing paper, the other mineral deposit locations were then digitized. This approach was repeated using all mineral deposit points as origin while maintaining the orientation of the tracing paper. From the Fry plots, a rose diagram was constructed to determine the orientation of mineral deposits within the study area.

### 4.2 Distance correlation analysis

Establishing a proximal relationship between mineral deposit occurrence and a set geological structure can be accomplished using a plethora of spatial and statistical tools [57, 62, 63]. Amongst these methods, the distance correlation analysis remains the most complementary for establishing spatial relationship between mineral deposits and specific geological structures. According to Carranza and Hale [62], and Carranza [58], the distance correlation analysis can be implemented using a binary curve that compares the cumulative frequency distribution of distances from a set of geological structures to known mineral occurrence and the cumulative frequency distribution of distances from a set of geological structures to non-deposit locations. Based on this plot, a positive certainty for control of mineralisation by specific geological structures are revealed by the occurrence of the distances to deposit plot above the distances to non-deposit plot as suggested by Carranza [58].

### 4.3 Prediction area plot analysis

Optimal prediction via application of knowledge driven predictive models are usually influenced by sufficient information on spatial association between exploration data and known mineral deposit occurrence. In most cases, the prediction ability for every spatial data is computed as weight values using a variety of statistical tools including Shannon entropy, information value, Chi-Square analysis or prediction area plot. The prediction area plot first proposed by Yousefi and Carranza [64], have been proven invaluable for establishing a spatial correlation between geospatial data and known mineral occurrence. The implementation of the analysis involved a comparative assessment of a cumulative area plot for mineral deposits and an inverse cumulative area plots for non-mineral deposit occurrences [65]. The intersection points between these two plots corresponds to its prediction accuracy.

### 4.4 TOPSIS

The TOPSIS model initially developed by Hwang and Yoon [66], can be regarded as a multi criteria approach with extensive applications in mineral predictive mapping [26, 27, 67, 68, 69]. Usually, it is considered a simplistic knowledge driven model due to its ability in handling problems that relates to significant number of alternatives [70]. According to Wang*, et al.* [71], the core principles of the TOPSIS model are based on generating a minimum distance between all options and the positive ideal solution, while maintaining a maximum distance to the negative ideal solution. Generally, the positive ideal solution optimises the cost criteria while the negative ideal solution maximises the cost criteria at the expense of minimising the cost benefits [72]. The systematic implementation of TOPSIS model involves a six step procedure [73].

**Step 1**: Generation and creation of a decision matrix as shown in Eq. (1).

(1) $$\begin{array}{ll} \; & \begin{array}{lll} F_{1} & F_{2} & \begin{array}{lll} \ldots \ldots \ldots . & F_{j} & \begin{array}{ll} \ldots \ldots \ldots . & F_{n} \end{array} \end{array} \end{array}\\ \begin{array}{l} A_{1}\\ A_{2}\\ \begin{array}{l} \cdot \\ \cdot \\ A_{J} \end{array} \end{array} & \left[\begin{array}{lll} \begin{array}{l} f_{1i}\\ f_{2i}\\ \begin{array}{l} \cdot \\ f_{j1}\\ f_{j2} \end{array} \end{array} & \begin{array}{l} f_{12}\\ f_{22}\\ \begin{array}{l} \cdot \\ f_{j2}\\ f_{j2} \end{array} \end{array} & \begin{array}{lll} \begin{array}{l} \ldots \ldots \ldots .\\ \ldots \ldots \ldots .\\ \begin{array}{l} \ldots \ldots \ldots .\\ \ldots \ldots \ldots \\ \ldots \ldots \ldots \end{array} \end{array} & \begin{array}{l} f_{1j}\\ f_{2j}\\ \begin{array}{l} \cdot \\ f_{ij}\\ f_{jj} \end{array} \end{array} & \begin{array}{l} \begin{array}{ll} \ldots \ldots & f_{1n} \end{array}\\ \begin{array}{ll} \ldots \ldots & f_{2n} \end{array}\\ \begin{array}{l} \begin{array}{ll} \ldots \ldots & \cdot \end{array}\\ \begin{array}{ll} \ldots \ldots . & f_{jn} \end{array}\\ \begin{array}{ll} \ldots .\ldots & f_{jn} \end{array} \end{array} \end{array} \end{array} \end{array}\right] \end{array}$$

**Step 2**: Calculation of a normalised decision matrix using the formula in Eq. (2).

(2) fij=fij∑j=1,jfij,2j=1,….j,i=1,…….n

**Step 3**: Calculation of weight normalised value calculated using the formula in Eq. (3).

(3) $$V_{ji}=W_{ij}r_{ij},\;j=1,\ldots \ldots \ldots j,\;i=1,\ldots \ldots ,\;n$$

Where the weight of the attribute or criterion and $\sum_{i=1}^{n}W_{i}=1$

**Step 4**: Determination of ideal and negative ideal solution (equation (4) and equation (5)).

(4) $$A^{+}=\left\{v_{1}^{+},\ldots \ldots \ldots .v_{n}^{+}\right\}=\left\{\left(maxv_{ij}/i\in 1^{\prime }\right),\left(minv_{ij}/i\in 1^{"}\right)\right\}$$

(5) $$A^{-}=\left\{v_{1}^{-},\ldots \ldots \ldots .v_{n}^{-}\right\}=\left\{\left(maxv_{ij}/i\in 1^{\prime }\right),\left(minv_{ij}/i\in 1^{"}\right)\right\}$$

Where $A^{+}$ is associated with the benefit criteria and $A^{-}$ is associated with the cost criteria.

**Step 5**: Consist of:

i) separation of each alternative from the positive ideal solution using Eq. (6).

(6) $$D_{j}^{+}=\sqrt{\overset{n}{\underset{i=1}{\sum }}{\left(v_{ij}-v_{i}^{+}\right)}^{2}\text{j ​}=\text{ ​}1\text{, ​}2\cdots \cdots \text{j}}$$

ii) separation of each alternative from the negative ideal solution using Eq. (7).

(7) $${D_{j}^{-}=\sqrt{\overset{n}{\underset{i=1}{\sum }}(v_{ij}-v_{i}^{-}})}^{2}\text{ ​ ​j}=1,2.........\text{j}$$

**Step 6**: Consist of calculating the relative closeness to the ideal solution as well as ranking the performance order using Eq. (8).

(8) $$CC^{+}=\frac{D_{J}^{-}}{D_{J}^{+}+D_{J}^{-}},\;J=1,2\ldots \ldots \ldots j,\;CC_{J}^{+}\in \left[0,1\right]$$

### 4.5 ARAS

The ARAS model first proposed by Zavadskas and Turskis [74] represents a multi-criteria method for optimally evaluating the significance of different alternatives within a given criteria. According to Petrović*, et al.* [75], the ARAS method is more specific and can evaluate the performance of every alternative by comparison to the optimal alternative. The implementation of the method begins with the development of a decision matrix followed by the generation of a weighted criteria. Usually, the following steps are applied for complete implementation of ARAS in decision making process.

**Step 1**: Determination of optimal performance rating for every criterion. This step is usually applied when the decision making does not have a preference. Thus, the optimal performance is computed using Eq. (9).

(9) $$X_{0i}\left\{ \begin{array}{l} max_{i}\;x_{ij}\;J\in \Omega_{max}\;\\ min_{i}\;x_{ij}\;\;j\in \Omega_{min} \end{array}\right.$$

$X_{0i}$ from Eq. (10) represents the optimal performance of the j−th ​criterion, $\Omega_{max}$ is the benefit criteria while $\Omega_{min}$ is the cost criteria.

**Step 2**: Consists of calculating the normalised decision matrix using Eq. (10).

(10) $$r_{ij}=\left\{ \begin{array}{l} \frac{x_{ij}}{\sum_{i=0}^{m}x_{ij}}\;:j\in \Omega_{max}\\ \frac{\frac{1}{x_{ij}}}{\sum_{i=0}^{m}\frac{1}{x_{ij}}}\;:j\;\in \Omega_{min} \end{array}\right.$$

Where $r_{ij}$ represents the normalised performance rating of the i−thalternatives to the j−th criterion i−0,1………m.

**Step 3**: Involves computing a weighted normalised decision matrix using the formula on Eq. (11).

(11) $$v_{ij}=w_{j}r_{ij}$$

Where $v_{ij}$ is considered the weighted normalised performance rating of the i−th to the j−th criterion i−0,1………m.

**Step 4**: Consist of calculating the overall performance rating for every alternative within a given criteria. The application of Eq. (12) can be a viable formula for this computation.

(12) $$S_{i}=\overset{n}{\underset{j=1}{\sum }}v_{ij}$$

$S_{i}$ represents the overall performance rating of the i−th alternatives i−0,1………m.

**Step 5**: The degree of unity for every alternative is calculated using the formula on Eq. (13).

(13) Qi=SiS0

$Q_{i}$ is the degree of unity of the i−th alternatives and also the general performance index of the optimal alternatives, i=1,2………m.

**Step 6**: Consist of ranking the various alternatives and selecting the most effective ones. Considered alternatives are ranked in ascending order based on their computed $Q_{i}$ values. Thus, the most acceptable alternatives are determined using the formula in Eq. (14).

(14) A∗={AimaxQii}

Where $A^{*}$ represents the most acceptable alternatives i=1,2………m.

### 4.6 MOORA

Multi-criteria decision making with the MOORA technique is generally considered an invaluable tool for optimising the best alternatives and identifying the most viable substitute within a given number of options [76, 77]. It generally attains a higher degree of efficiency in the rating and selection of alternatives as it is virtually free of complexity [78]. According to Ajrina*, et al.* [79], the enhanced selective proficiencies of the MOORA model can be attributed to its capability in identifying and differentiating the various conflicting criteria into beneficial (maximising) and non-beneficial (minimising). This process eliminates the inappropriate criteria and strengthens the selecting process [76]. Statistically, the MOORA model can be implemented through a given number of steps.

**Step 1**: Involves the creation and generation of a decision matrix designated by an $X_{ij}$ matrix as shown in Eq. (15).

(15) $$X_{\;}=[\begin{array}{lll} X_{11} & X_{12} & .\\ X_{21} & X_{22} & .\\ X_{m1} & X_{m2} & . \end{array}\begin{array}{l} X_{1n}\\ X_{2n}\\ X_{mn} \end{array}]$$

i represents the m number of alternatives, while j is the n number of criteria.

**Step 2**: Involves the normalisation of the decision matrix using the formula computed in Eq. (16).

(16) Xij∗=Xij[∑i=1mXij2](j=1,2………..n)

**Step 3**: Consists of optimising the different attributes by adding the normalised performance in case of maximisation (for favourable attributes) and deduction in case of minimisation (for non-beneficial attributes). The optimisation of different criteria is implemented using the formula in Eq. (17).

(17) $$Y_{i}=\overset{g}{\underset{j=1}{\sum }}X_{ij}^{*}-\overset{n}{\underset{j=g+1}{\sum }}X_{ij}^{*}$$

Where g within this equation represents the number of attributes to be maximised, while n−g represents the number of attributes to be minimised. $Y_{i}$ is the resultant normalised value of all alternatives within every attribute.

**Step 4**: Because some attributes are usually more important in predicting a given event, it is essential to incorporate a weight factor illustrated in Eq. (18).

(18) $$Y_{i}=\overset{g}{\underset{j=1}{\sum }}W_{j}X_{ij}^{*}-\overset{n}{\underset{j=g+1}{\sum }}W_{j}X_{ij}^{*}\;\left(j=1,2\ldots \ldots \ldots \ldots ,n\right)$$

The output value $Y_{i}$ is usually positive or negative depending on its maximal (favourable attribute) and minimal (unfavourable attribute) number. The ordinal ranking of $Y_{i}$ is an indicator of its resultant preference.

### 4.7 Receiver Operating Characteristic Curve/Area Under Curve

Performance assessment of every GIS model is generally considered invaluable in predicting future events [80, 81] as it guarantees an augmented certainty in decision making processes. The Receiver Operating Characteristic Curve and Area Under Curve (ROC/AUC) analysis are considered a more robust approach for evaluating accuracy of GIS predictive models [82], as it exhibit an independent effect from multiple threshold values [83]. Basically, the ROC represents a binary plot that assesses and compares the true positive rate (Sensitivity) to the false positive rate (1-Specificity) at various thresholds intervals. It evaluates the performance of every GIS model via a binary classification system alongside continuous variable [84]. Usually, model efficiency is often ranked from 0-1 using the AUC output. Values closer to 0 are indicative of poor performance for the predictive model while those closer to 1 are indications of excellent performance [85]. The ROC and AUC techniques were used for quantitative and qualitative evaluation of knowledge driven predictive models for gold mineralisation.

## 5 Results and discussion

**Figure 7:**
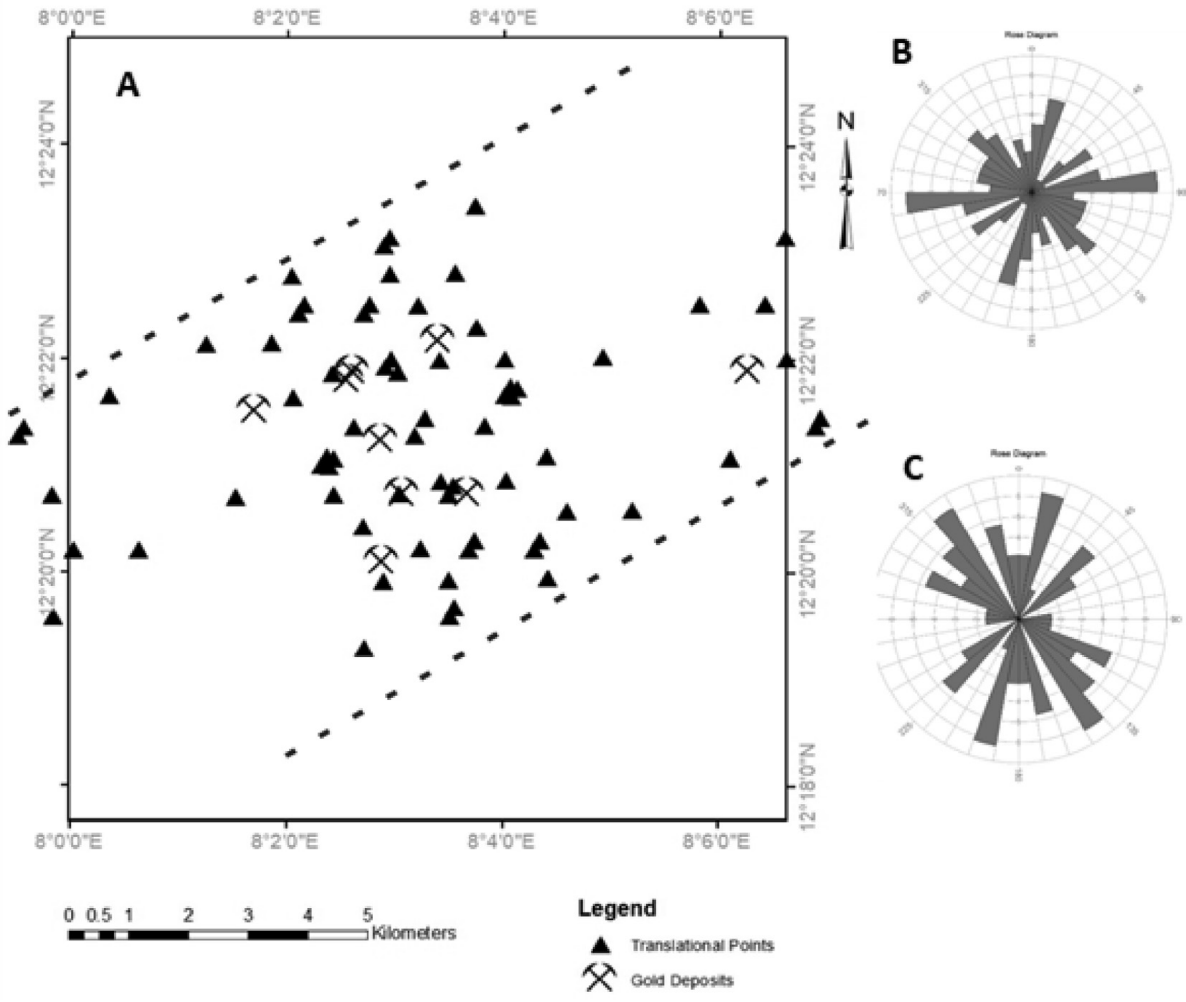
Structural evaluation using Fry analysis: (a) Translational points; (b) Rose plots for all points; (c) Rose plots for points, 3 km apart.

**Figure 8:**
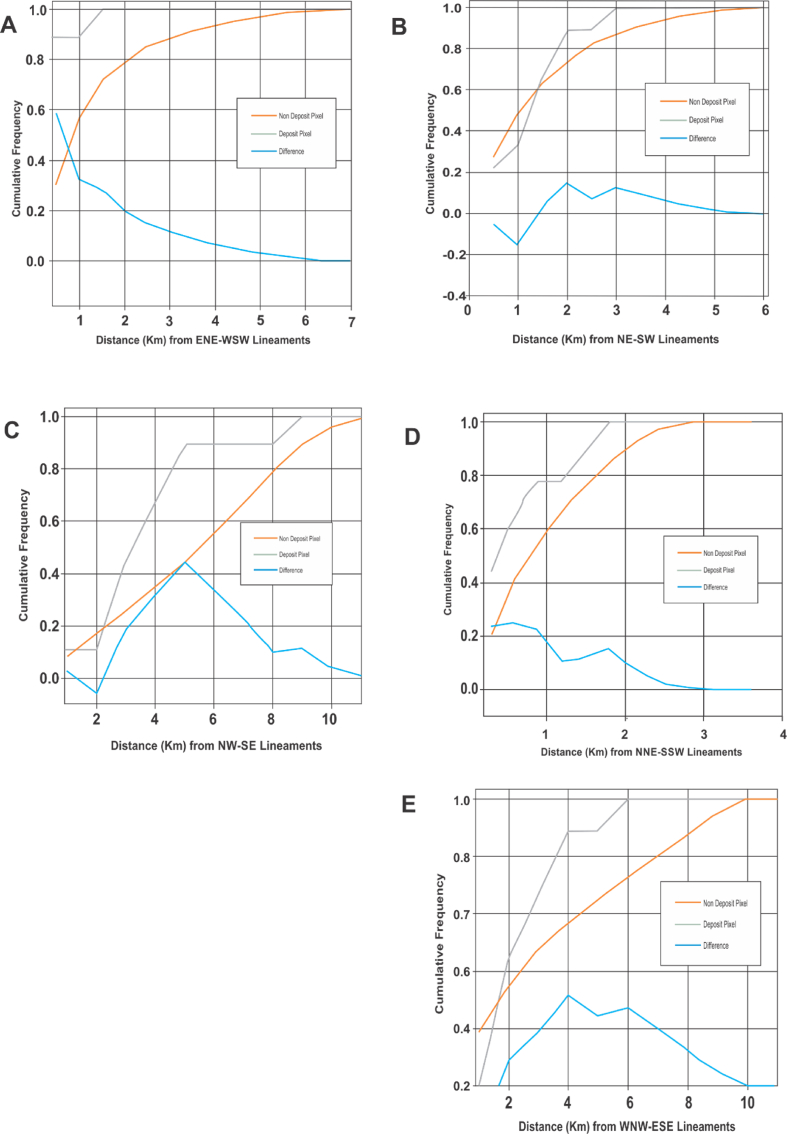
Distance correlation analysis for lineaments: (a) ENE-WSW lineaments; (b) NE-SW lineaments; (c) NW-SE lineaments; (d) NNE-SSW lineaments; (e) WNW-ESE lineaments.

The effective structural control for gold mineralisation within the Malumfashi area is illustrated using the Fry plot and distance correlation analysis plots as shown in Figures 7 and 8. Based on the Fry analysis, gold mineralisation within the Malumfashi area displayed a dominant E-W trending direction as well as a less dominant NE-SW and NW-SE directions. Also, at a more localised distance of 3 km, the Fry plot suggests a significant affiliation to the NNE-SSW, NE-SW and NW-SE trends. The evidence from distance correlation analysis reveals a more proximal relationship of gold occurrence to the ENE-WSW and NNE-SSW directions. No discrete affiliation or positive spatial correlation was observed between gold occurrence and NE-SW, NW-SE, and WNW-ESE lineament types (Figure 8b, c and e). The positive spatial association observed between gold deposits with the ENE-WSW lineaments is characterised by an optimal spatial association at a distance of 0.5 km, and within this distance, 88.9% of all known mineral deposits are present and there is a 60% chance higher than normal for finding mineralisation. More so, the NNE-SSW lineaments are characterised by an optimal spatial association at distances of 0.6 km. Within this distance, 0.75% of all known mineral deposits are present and there is a 0.25% chance higher than normal for finding mineralisation.

A statistical correlation analysis amongst predictive variables is shown in Table 2. Evidence from the Pearson correlation matrix suggest a generally low correlation amongst all spatial predictive variables. A more significant negative correlation (−0.6) is observed between the first vertical derivative variable and lineament density (Table 2).

**Table 2:** Statistical correlation analysis for exploration data.

	HR	NNE-SSW	ENE-WSW	LIN DEN	FERRIC	FERROUS
NNE-SSW	-0.28					
ENE-WSW	-0.18	-0.31				
LIN DEN	0.22	0.53	0.00			
FERRIC	0.34	0.25	-0.24	0.40		
FERROUS	0.26	0.04	-0.06	-0.32	0.20	
FVD	-0.07	-0.06	0.20	-0.62	-0.41	0.43

**Figure 9:**
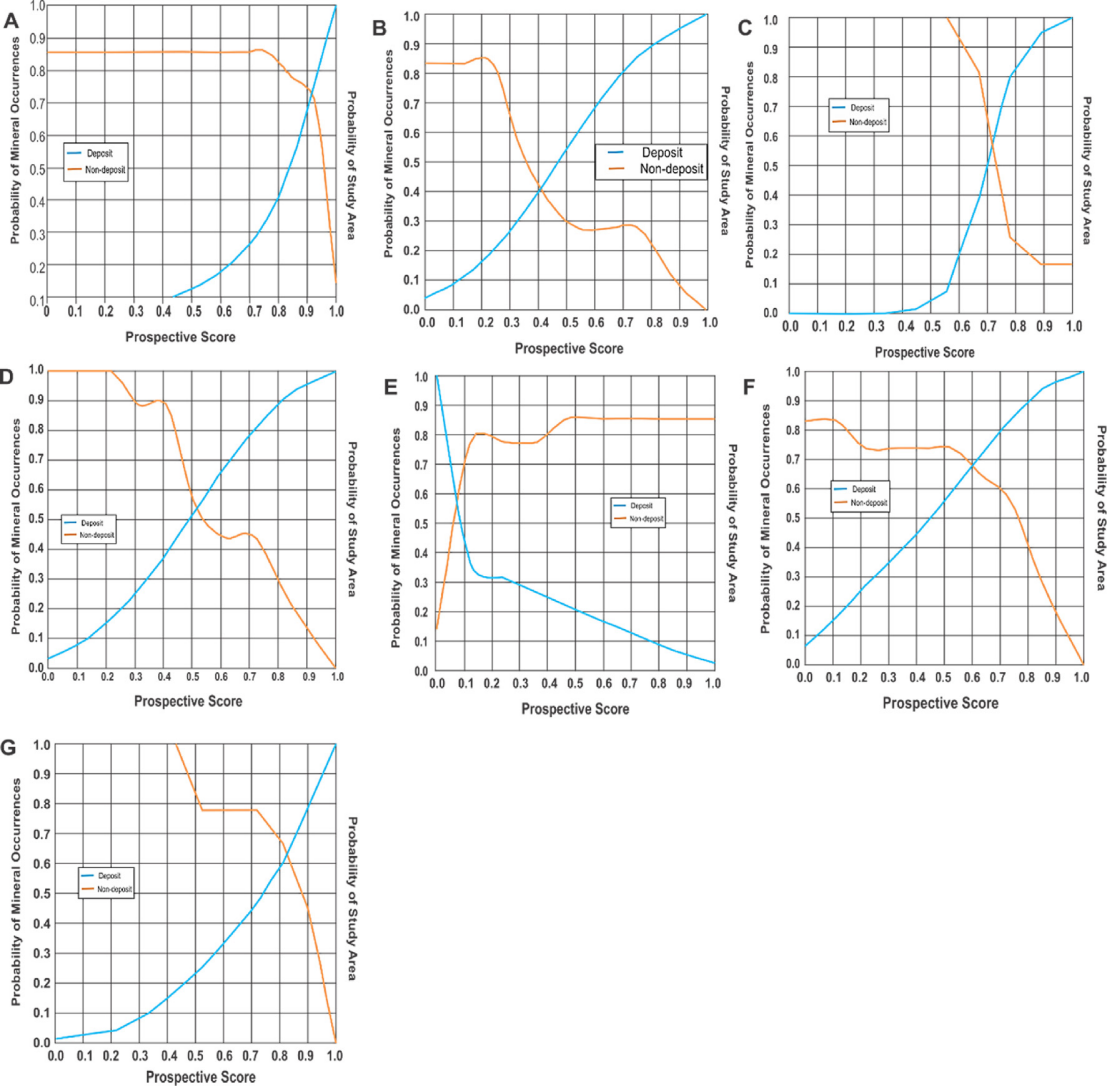
Analysis of spatial correlation of mineral deposits with exploration dataset: (a) ENE-WSW Lineaments; (b) Ferric iron; (c) First vertical derivatives; (d) Ferrous iron; (e) Host rock; (f) Lineament density; (g) NNE-SSW lineaments.

Figure 9 illustrates a statistical correlation analysis between known gold occurrence and spatial predictive datasets. Analysis of these plots suggest a relative significant spatial association between gold mineralisation with structural dataset defined by correlation levels above 60%. The least correlation relationship with gold mineralisation were obtainable from spatial data pertaining to alteration information with correlation levels of 0.54 (ferrous iron alteration) and 0.41 (ferric iron alteration). A statistical summary for spatial data correlation with known mineralisation is shown in Table 3.

**Table 3:** Computed weights extracted from the prediction area plots.

SN	Data	Weight	Mean	STD
1	Lineament Density	0.68	0.59	0.1017
2	Distances to ENE-WSW Lineaments	0.72		
3	Distances to NNE-SSW Lineaments	0.63		
4	Distances to Host Rock	0.57		
5	First vertical Derivatives	0.58		
6	Ferrous Iron alteration	0.54		
7	Ferric Iron alteration	0.41		

**Figure 10:**
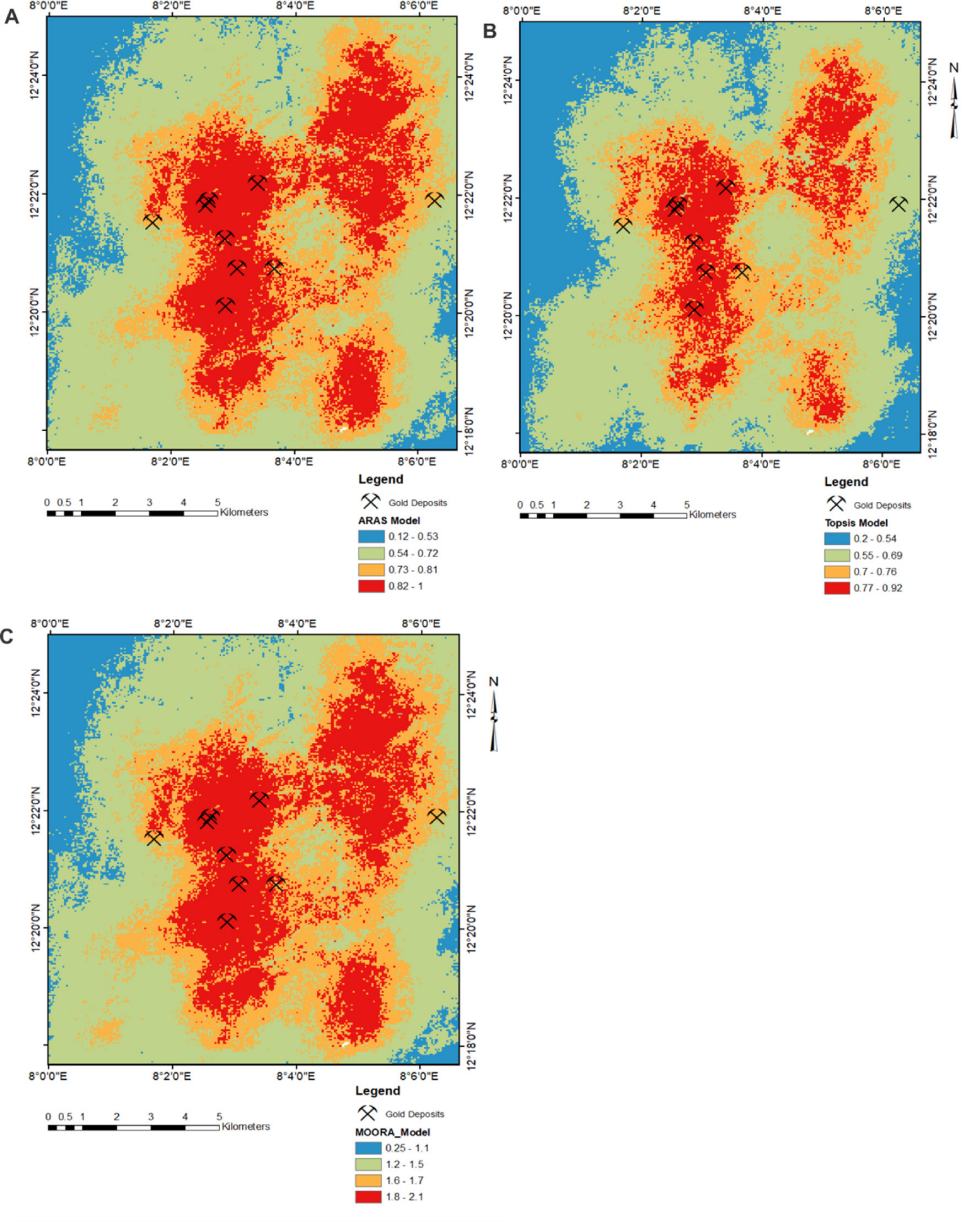
Gold predictive models for parts of the Malumfashi area showing: (a) Application of the ARAS model; (b) Application of the TOPSIS model; (c) Application of the MOORA model.

**Figure 11:**
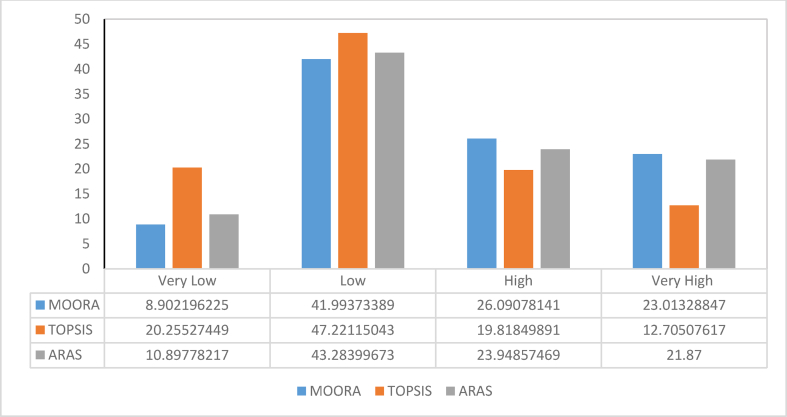
Statistical distribution of predictive classes across all models.

A total of seven datasets consisting of geology, geophysical, structural, and remote sensing data were effectively integrated for gold predictive mapping using three multi-criteria decision models consisting of TOPSIS, ARAS and MOORA (Figure 10). Spatial segregation into four classes using the natural break interval method reveals a significant favourability for gold mineralisation within the southern, central, and north-eastern parts of the study area. The least favourable zones occupy the western and north-western axis of the study location. A statistical assessment of percentile extent for every predictive class within each model is shown in Figure 11. Based on the diagram, the highly favourable zones within the TOPSIS model occupy 12.7% of the study area. The highly favourable zones within the ARAS and MOORA models accounted for 21.8% and 23.0%, respectively. The high potential class within the TOPSIS model appears to occupy a percentile extent of 19.8%. This is slightly lower when compared to the same class on the ARAS and MOORA models with percentile extents of 23.9% and 26.0%, respectively. The low prospective classes for these models are characterised by a percentile distribution of 47.2% for TOPSIS model, 43.2% for ARAS and 41.9% for the MOORA model. The very low prospective zones are defined by a percentile extent of 20.2% (TOPSIS), 10.8% (ARAS) and 8.9% (MOORA), respectively (Figure 11).

**Figure 12:**
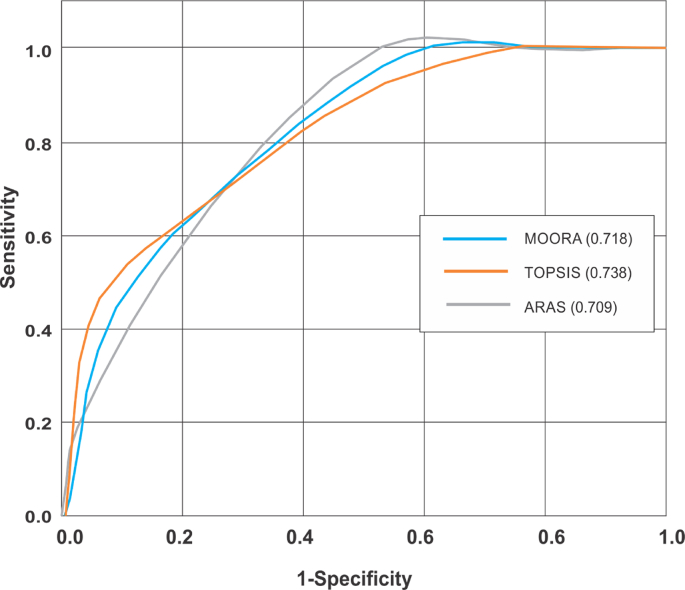
Evaluation of model accuracy and efficiency using the ROC/AUC plots.

A visual analysis of the spatial association between known gold occurrence and every predictive model suggests a significant association with zones of high favourability. Within these models, approximate estimate of 77% of all gold mineralisation appears to fall within the most favourable predicted zones. The statistical validation using the ROC/AUC tool shown in Figure 12 suggest a significant reliability for all models with prediction accuracies above 70%. A comparative assessment of prediction accuracy for these models suggests the TOPSIS model had the best prediction efficiency defined by prediction levels of 73.8%. This was closely followed by the MOORA model with prediction accuracy of 71.8%. The ARAS model had the least prediction efficiency characterised by prediction accuracy of 70.9%.

Geological evidence from several studies in the open literature [21, 52, 86, 87] have revealed a structural attribute for primary gold mineralisation within the Nigerian basement complex. The structural styles associated with this mineralisation type is generally considered viable evidence for the implementation of Fry and distance correlation analysis in evaluating structural attributes for gold prospects within the Malumfashi area. Based on Fry analysis, known gold occurrence within the study location assumed a distinct E-W and NE-SW pattern accompanied by a less prominent but significant NW-SE affiliation. The deposit scale structural analysis at less than 2 km suggests a predominant NE-SW and NW-SE affiliations. The application of the distance correlation analysis suggest gold mineralisation within this location is more associated with the ENE-WSW structures, with a slight positive spatial association observed for the NNE-SSW lineaments. The discrepancy in results obtained from the Fry and distance correlation methods can be attributed to inconsistency in sampling for gold occurrence across the study area.

Bonham-Carter [88] have suggested the distance to structure analysis is a more representative approach for interpreting the regional structural attributes of mineral deposits since a higher intensity is likely at proximal distances. The close association of gold mineralisation with the NNE-SSW trends have been observed within the Nigerian schist belts [20, 21] and have been linked to the Pan-African orogenic event [89]. The ENE-WSW proximity of gold deposits remains uncommon, but the structural trend have been reported as being of Kibaran orogenic event [90].

The application of multi-criteria methods for predicting mineral deposit occurrence are well suited for green field programs [16]. In most cases, an expert opinion is required for adequate assignment of weighted values to different classes within each variable [68]. For effective implementation of this model, a statistical correlation test was carried out to identify and eliminate data redundancy. The generally low correlation (<0.7) amongst all predictor variables suggests the absence of data redundancy and validates their effective integration for generating mineral predictive maps [91]. An augmented analysis using the prediction area plots was very effective in evaluating the prediction ability of different spatial data and served as a weighted value for optimising the knowledge driven models [64,65,92]. Within the Malumfashi area, a comparative assessment of three multi-criteria models (TOPSIS, ARAS and MOORA) were made and spatial evidence from these models suggest a high potential for gold mineralisation is more likely to occur in southern, central, and north-eastern part of the study area. A statistical estimate of these models suggests the most favourable zones for gold mineralisation is more significant for the MOORA (23.01%) and ARAS (21.87%) models when compared to the TOPSIS (12.7%) model. The TOPSIS model appears to have a higher degree of prediction accuracy for gold occurrence when compared to the MOORA and ARAS models.

## 6 Conclusions

Regional structural mapping and data integration methods are important tools for mineral exploration at regional extents. The assessment of prediction competence for every geospatial data and the incorporation of derived weights into GIS models have been invaluable in augmenting prediction accuracy of multi-criteria models. Positive spatial association of gold deposits with the NNE-SSW as well as the ENE-WSW may suggest the genesis of gold mineralisation involved two distinct orogenic episodes. The close spatial similarity amongst the TOPSIS, MOORA and ARAS models as well as the high prediction accuracy above 70% are reliable indicators that these models could be effective in prospecting gold mineralisation within the study area.

## Declarations

### Author contribution statement

Andongma W. Tende: Conceived and designed the experiments; Performed the experiments; Analyzed and interpreted the data; Contributed reagents, materials, analysis tools or data; Wrote the paper.

Mohammed D.Aminu, Abdulgafar K. Amuda and Jiriko N. Gajere: Analyzed and interpreted the data; Contributed reagents, materials, analysis tools or data.

Hadiza Usman and Fatima Shinkafi: Analyzed and interpreted the data.

### Funding statement

This research did not receive any specific grant from funding agencies in the public, commercial, or not-for-profit sectors.

### Data availability statement

Data will be made available on request.

### Declaration of interests statement

The authors declare no conflict of interest.

### Additional information

No additional information is available for this paper.
